# Vitamin E stabilizes iron and mitochondrial metabolism in pulmonary fibrosis

**DOI:** 10.3389/fphar.2023.1240829

**Published:** 2023-12-06

**Authors:** Jing Chang, Jiahui Wang, Beibei Luo, Weihao Li, Ziyue Xiong, Chaoqi Du, Xue Wang, Yuejiao Wang, Jingya Tian, Shuxin Li, Yue Fang, Longjie Li, Jing Dong, Ke Tan, Yumei Fan, Pengxiu Cao

**Affiliations:** ^1^ Ministry of Education Key Laboratory of Molecular and Cellular Biology, Hebei Key Laboratory of Animal Physiology, Biochemistry and Molecular Biology, Hebei Research Center of the Basic Discipline of Cell Biology, College of Life Sciences, Hebei Normal University, Shijiazhuang, Hebei, China; ^2^ Special Clinical Laboratory, The Second Hospital of Hebei Medical University, Shijiazhuang, Hebei, China

**Keywords:** pulmonary fibrosis, vitamin E, iron, mitochondria, fibroblast

## Abstract

**Introduction:** Pulmonary fibrosis (PF) is a fatal chronic lung disease that causes structural damage and decreased lung function and has a poor prognosis. Currently, there is no medicine that can truly cure PF. Vitamin E (VE) is a group of natural antioxidants with anticancer and antimutagenic properties. There have been a few reports about the attenuation of PF by VE in experimental animals, but the molecular mechanisms are not fully understood.

**Methods:** Bleomycin-induced PF (BLM-PF) mouse model, and cultured mouse primary lung fibroblasts and MLE 12 cells were utilized. Pathological examination of lung sections, immunoblotting, immunofluorescent staining, and real-time PCR were conducted in this study.

**Results:** We confirmed that VE significantly delayed the progression of BLM-PF and increased the survival rates of experimental mice with PF. VE suppressed the pathological activation and fibrotic differentiation of lung fibroblasts and epithelial-mesenchymal transition and alleviated the inflammatory response in BLM-induced fibrotic lungs and pulmonary epithelial cells *in vitro*. Importantly, VE reduced BLM-induced ferritin expression in fibrotic lungs, whereas VE did not exhibit iron chelation properties in fibroblasts or epithelial cells *in vitro*. Furthermore, VE protected against mitochondrial dysmorphology and normalized mitochondrial protein expression in BLM-PF lungs. Consistently, VE suppressed apoptosis in BLM-PF lungs and pulmonary epithelial cells *in vitro*.

**Discussion:** Collectively, VE markedly inhibited BLM-induced PF through a complex mechanism, including improving iron metabolism and mitochondrial structure and function, mitigating inflammation, and decreasing the fibrotic functions of fibroblasts and epithelial cells. Therefore, VE presents a highly potential therapeutic against PF due to its multiple protective effects with few side effects.

## 1 Introduction

Idiopathic pulmonary fibrosis (IPF) is a fatal disease characterized by the loss of respiratory lobules caused by fibrosis and the remodeling of alveolar units, which leads to a decline in pulmonary function. Many factors, including susceptible genetic factors, viral infection, radiation, and smoking, may cause IPF (Cong et al., 2017). The only two drugs approved by the FDA for IPF, pirfenidone and ninedanib, slow disease progression in some IPF patients but have little impact on the survival rate of IPF patients and have some side effects (King et al., 2014; Richeldi et al., 2014; Rangarajan et al., 2016). At present, lung transplantation is the only therapeutic strategy that significantly prolongs the survival of severe IPF patients, but the probability of pulmonary fibrosis (PF) caused by allograft rejection is also high. Therefore, it is urgent to identify effective therapeutic strategies for PF.

In recent years, it has been reported that pulmonary iron accumulation and dysregulated iron homeostasis are associated with or promote the progression of many lung diseases, such as IPF, chronic obstructive pulmonary disease, cystic fibrosis, alveolar proteinosis, lung cancer, asthma and acute lung injury (Ghio et al., 2008; Ali et al., 2017; Cloonan et al., 2017; Neves et al., 2019; Zhang et al., 2019; Zhu et al., 2021). Accumulated iron generates excessive ROS through the Fenton reaction, which causes functional changes in cells, such as inflammation or ferroptosis mediated by lipid peroxidation (Kajarabille and Latunde-Dada, 2019; Battaglia et al., 2020). Because the lung contacts the external environment directly, maintaining pulmonary iron homeostasis is a major challenge. The regulation of pulmonary iron homeostasis mainly depends on iron regulatory molecules, such as ferritin, transferrin receptor (TFRC), divalent metal ion transporter (DMT1), and ferroportin 1 (FPN1). Cytosol iron is either wrapped by ferritin, thus reducing iron toxicity to cells, forms a liable iron pool (LIP) or is relocated to mitochondria to form iron sulfur clusters and heme. TFRC mediates the endocytosis of Fe^3+^ bound to transferrin (TF). DMT1 transports Fe^2+^ from the extracellular space to the cytosol, and FPN1 pumps iron out of the cell. It has been reported that pulmonary levels of iron and ferritin are elevated in bleomycin (BLM)-induced fibrotic mouse lungs, and the levels of iron regulatory proteins are also altered (Zhu et al., 2021).

Aging is one of the predisposing factors of IPF. With aging, mitochondria show abnormal morphology and structure, such as swelling, the disappearance of cristae, and the rupture of the intima (Mora et al., 2017). Importantly, oxidative stress dysregulates mitochondrial dynamics, such as mitochondrial fission and fusion imbalance, thus affecting the shape, size and number of mitochondria, mitochondrial dysfunction and subcellular transport, further affecting a variety of cell functions and even causing cell death and a variety of human diseases (Yu et al., 2020). In the lungs of IPF patients and BLM-PF lungs, mitochondrial morphology and number were dysregulated, and mitochondrial dysfunction occurred (Bueno et al., 2015; Rangarajan et al., 2018; Yu et al., 2018).

Vitamin E (VE) is a group of antioxidant liposoluble vitamins, including α-, β-, γ-, and δ-tocopherol and α-, β-, γ-, and δ-tocotrienol; α-tocopherol is the most biologically active form of VE (Abdala-Valencia et al., 2013). VE has been reported to attenuate BLM-induced PF in experimental animals in only a few reports, and its explored functional mechanisms are restricted to antioxidation and the inhibition of inflammation (Deger et al., 2007; Shi et al., 2013; Ramezani et al., 2021). In addition, VE supplementation immediately after irradiation was reported to protect against radiation-induced PF in rats (Bese et al., 2007). In recent years, iron metabolism and mitochondrial metabolism have received much attention and become the focus of pathogenesis studies on PF. However, the effects of VE on iron metabolism and mitochondrial structure and function in fibrotic lungs have not been explored. Therefore, in this study, we administered VE to experimental mice with PF or cultured pulmonary cells and investigated the unknown aspects of its functional mechanisms, especially whether the improvement of iron and mitochondrial metabolism plays a role in the inhibition of PF by VE.

## 2 Materials and methods

### 2.1 Establishment of the BLM-PF mouse model and oral gavage

All animal procedures complied with NIH guidelines for the Care and Use of Laboratory Animals and were approved by the Biomedical Ethics Committee of Hebei Normal University (approval Nos 2019SC02 and 2020LLSC017) and the Research Ethics Committee of the Second Hospital of Hebei Medical University (approval number: 2020-R331). Eight-week-old male C57BL/6J mice (Beijing Charles River, China) were adapted for 1 week, and randomly divided into 3 groups (PBS, BLM, and BLM + VE) or 2 groups (PBS and VE). After anesthetization with 5% chloral hydrate (5 mL/kg), 50 μL PBS with or w/o 1.5 U/kg BLM (B107423, Aladdin, Shanghai, China) was instilled to the corresponding animals through the trachea. The dose of VE was 200 mg/kg by oral gavage once daily, and the duration was indicated in different experiments.

### 2.2 Extraction of primary lung fibroblasts and cell culture

C57BL/6J male mice (4–6 weeks) were anesthetized with 5% chloral hydrate (10 mL/kg) by intraperitoneal injection, and then mouse lungs were immediately isolated, cut into small pieces and digested in serum-free medium containing 0.1% collagenase Ⅰ (C8140, Solarbio, Beijing, China) at 37°C in a shaker for 40 min. After filtration through a 70 μm strainer and centrifugation, the cell pellet was suspended and plated in cell culture dishes with high-glucose DMEM containing 10% FBS (FSP500, ExCell Bio, Shanghai, China), 100 U/mL penicillin, 0.1 mg/mL streptomycin, and 0.25 μg/mL amphotericin B at 37°C in a cell incubator with 5% CO_2_. Primary lung fibroblasts at passage 2 were utilized for the indicated treatments.

Mouse lung epithelial cell line MLE 12 was purchased from the American Type Culture Collection (ATCC) and cultured in DMEM: Ham’s F12 medium supplemented with 2% FBS, 0.01 mg/mL transferrin, 0.005 mg/mL insulin, 30 nM sodium selenite, 10 nM β-estradiol, 10 nM hydrocortisone, 2 mM L-glutamine and 10 mM HEPES at 37°C in an incubator with 5% CO_2_.

### 2.3 RNA extraction and real-time PCR

RNA extraction and quantitative real-time PCR were performed as described (Zhu et al., 2021). The primer sequences were provided in the online data supplement.

### 2.4 Immunoblot analysis

Immunoblotting was conducted as previously described (Zhu et al., 2021). The primary antibodies were described in online data supplement. The quantification of the target bands was performed by ImageJ, and the quantified results from different gels were combined after normalizing the average expression level of the target protein in all the control samples in one gel as 1.

### 2.5 Histology examinations

Analyses of three parameters for PF of the experimental mice were performed based on Masson-stained lung sections. Ashcroft score was analyzed as described (Hübner et al., 2008). Airway thickness was measured by Vvsub method with ImageJ software (Mimura et al., 2015). Collagen volume fraction was defined as the collagen area/total area of the tissues in the image and analyzed by ImageJ (Geng et al., 2022).

### 2.6 Immunofluorescent staining

Immunofluorescent staining was conducted as described (Zhu et al., 2021). The following antibodies were utilized: anti-Collagen I (1:100, GB11022-1, Servicebio, Wuhan, China), anti-Pdgfrα (1:100, Sc-21789, Santa Cruz Biotechnology, Dallas, United States), anti-BrdU (1:3,000, GB12051, Servicebio), anti-α-SMA (1:100, ARG66381, Arigo Biolaboratories, Shanghai, China), anti-Ftl (1:100, K002972P, Solarbio, Beijing, China), and anti-Cd68 (1:100, MAB5580, Millipore). Quantitative analyses of the immunofluorescent images were performed by ImageJ or as indicated in the corresponding figure legends.

### 2.7 Statistics

All data are presented as the mean ± SEM using GraphPad Prism 7.00. Two-tailed *t*-test was applied for comparisons between 2 groups. One-way ANOVA followed by a Bonferroni multiple comparisons test was conducted for 3 groups. *p* < 0.05 was considered statistically significant.

## 3 Results

### 3.1 VE improves BLM-induced PF and animal survival

**FIGURE 1:**
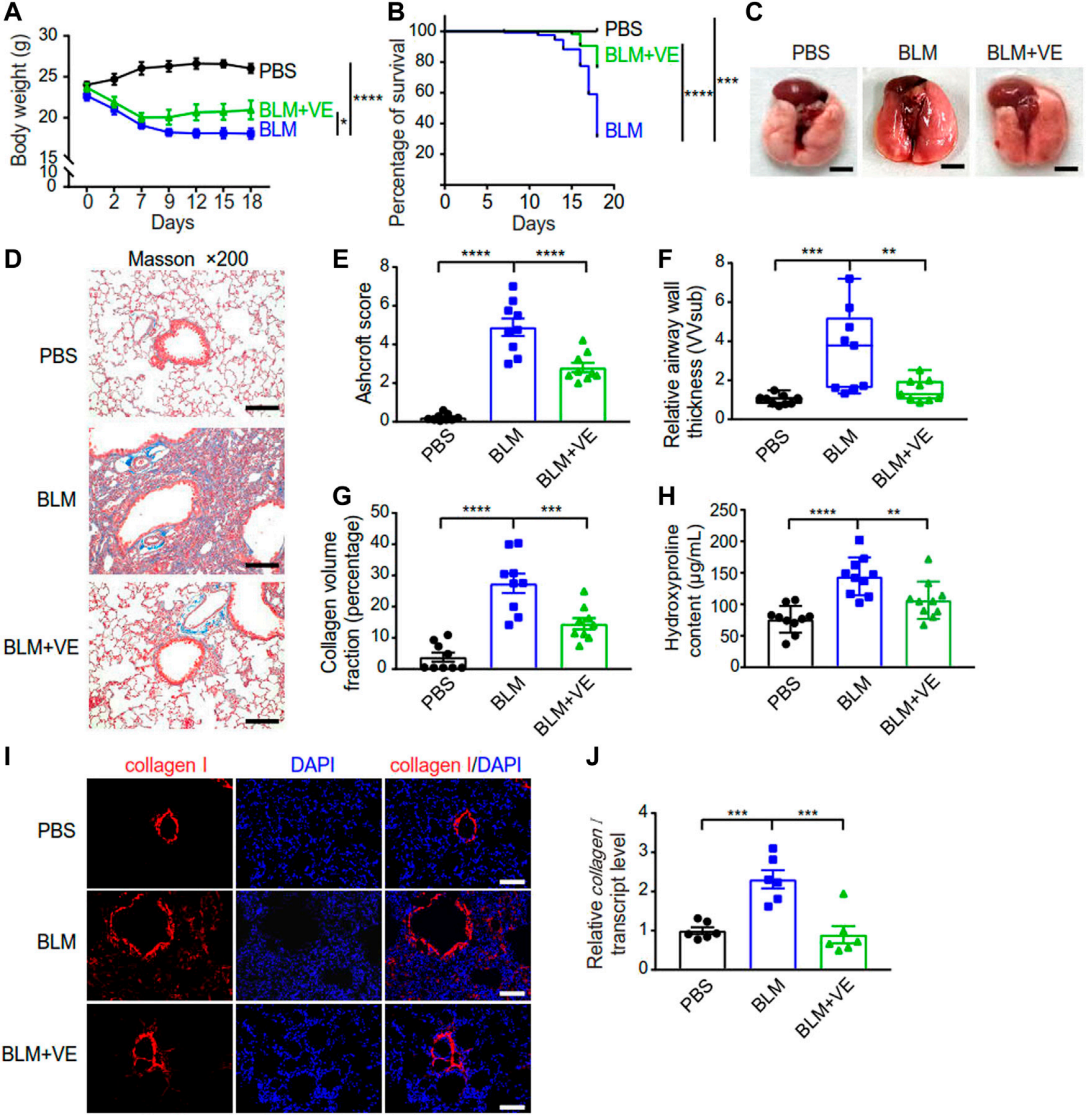
VE attenuated BLM-PF in experimental mice. VE administration was started from Day 7 till harvest on Day 18 post BLM instillation. **(A,B)** Changes in the body weight (A, *n = 8/group*) and survival rate (B, *n = 19/group*) of experimental mice during the time-course post BLM modeling. **(C)** Representative macroscopic appearance of mouse lungs as indicated. Scale bars, 0.5 cm. **(D)** Representative Masson images of mouse lung sections. Scale bars, 100 μm. **(E–G)** Ashcroft score (E, *n = 9/group*), relative airway wall thickness (F, *n =9/group*), and collagen volume fraction (G, *n = 9/group*) were analyzed based on pathological analysis of Masson images of mouse lung sections. **(H)** Hydroxyproline assay was performed to examine collagen content in mouse lungs. *n* = 10/group. **(I,J)** Representative immunofluorescent staining images **(I)** and transcript level of collagen I (J, *n = 6/group*) in mouse lung tissue detected by real-time PCR. Scale bars, 100 μm. Two-way ANOVA **(A)**, Log-rank (Mantel-Cox) test **(B)**, and One-way ANOVA **(E–H,J)**, *p < 0.05, **p < 0.01, ***p < 0.001, ****p < 0.0001.

To verify the inhibitory effect of VE on PF, we established a PF mouse model by administering BLM through the trachea, and mice instilled with PBS were the controls. Treatment with 200 mg/kg VE was applied correspondingly by gavage once daily starting on Day 7 after BLM instillation until the end of the experiment. VE treatment slowed weight loss by 28% (Figure 1A) and improved the survival rate by 51% at Day18 (Figure 1B) in bleomycin-induced pulmonary fibrosis (BLM-PF) mice. In addition, VE alleviated the macroscopic appearance of BLM-injured lungs (Figure 1C). Importantly, pathological analysis of Masson-stained lung sections in the VE-treated group showed a better Ashcroft score, decreased airway thickness, and less collagen volume fraction (Figures 1D–G). Consistently, VE-mediated suppression of collagen content in BLM-injured lungs was confirmed by hydroxyproline quantification and immunofluorescent staining (Figures 1H, I), which seems to be mediated by mRNA regulation (Figure 1J). Collectively, these results confirmed that VE attenuated BLM-induced PF, as evidenced by reduced extracellular matrix accumulation in fibrotic mouse lungs and increased animal survival rates.

### 3.2 VE regulates activation and fibrotic differentiation of fibroblasts and epithelial-mesenchymal transition (EMT)

**FIGURE 2:**
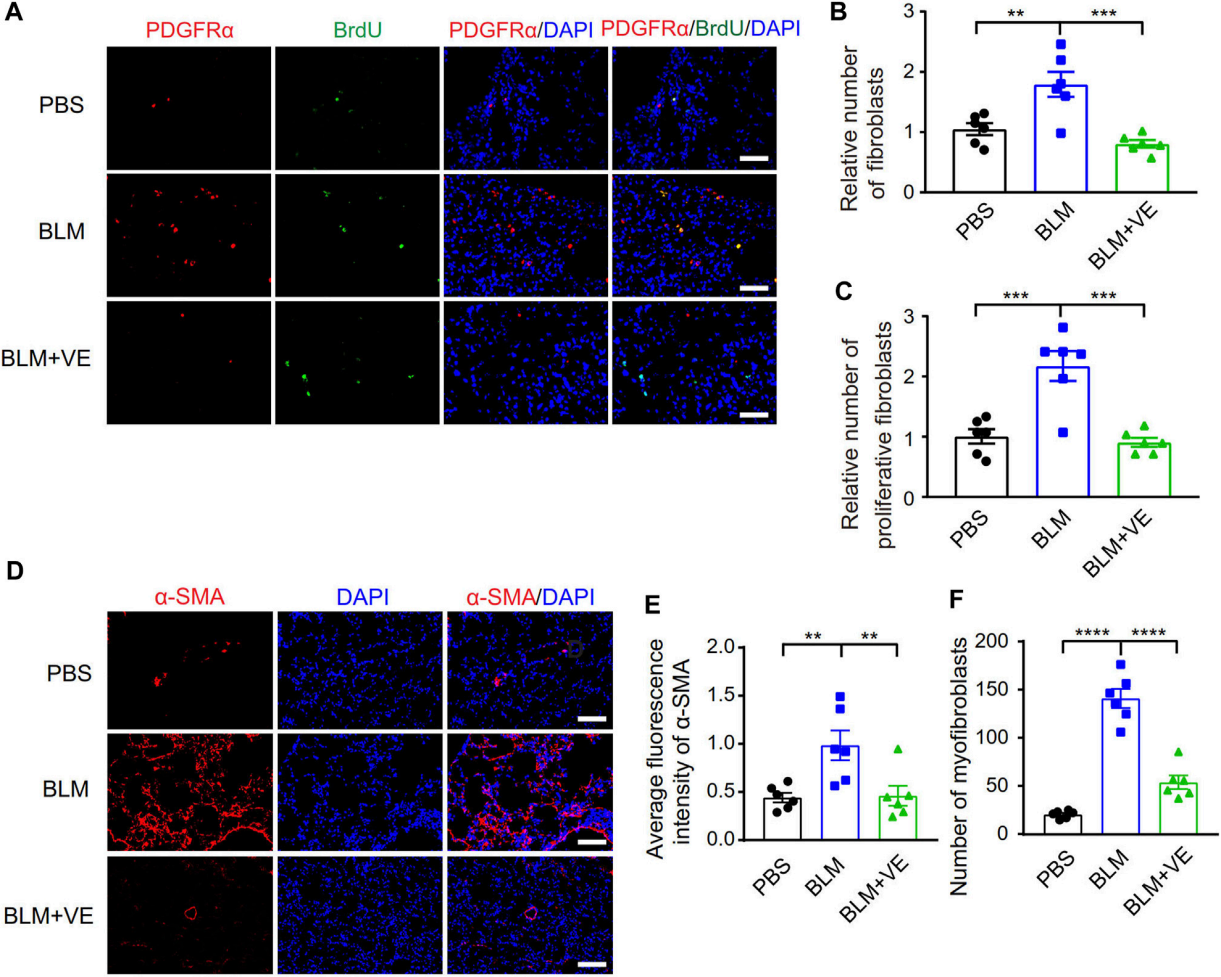
VE inhibited proliferation and fibrotic differentiation of fibroblasts in BLM-PF lungs. **(A–C)** Mouse lungs were instilled with 100 mg/kg 5-bromo-2′-deoxyuridine (BrdU) through trachea on Day 14 post BLM modeling, harvested 48 h later, and immunofluorescent staining was performed on lung sections **(A)**. Scale bars, 50 μm. The numbers of fibroblasts (Pdgfrα^+^) (B, *n = 6/group*) and proliferating fibroblasts (Pdgfrα^+^/BrdU^+^) (C, *n = 6/group*) were counted from three random immunofluorescent images per mouse, and the cell numbers were normalized to the PBS group. **(D)** Representative immunofluorescent staining images of lung sections from experimental mice on Day 18 post BLM injury. Scale bars, 100 μm. **(E,F)** Quantitative analysis (E, *n =6/group*) on the fluorescence intensity of α-SMA in ratio to that of DAPI was performed by ImageJ and the number of myofibroblasts (α-SMA^+^) (F, *n = 6/group*) in each microscopic image in mouse lungs was counted based on three random immunofluorescent images per mouse. One-way ANOVA, **p < 0.01, ***p < 0.001, ****p < 0.0001.

The activation and fibrotic differentiation of pulmonary fibroblasts are key factors affecting the production of extracellular matrix, and whether these processes are affected by VE has not been reported previously. We observed that VE suppressed the number of total fibroblasts (Pdgfrα^+^) and proliferating fibroblasts (Pdgfrα^+^/BrdU^+^) in BLM-PF mice, as shown by immunostaining (Figures 2A–C). Furthermore, VE significantly inhibited BLM-induced expression of smooth muscle actin α2 (α-SMA) and decreased the number of myofibroblasts (α-SMA^+^) (Figures 2D–F). Therefore, VE suppresses the activation and fibrogenesis of fibroblasts in BLM-PF lungs *in vivo*.

**FIGURE 3:**
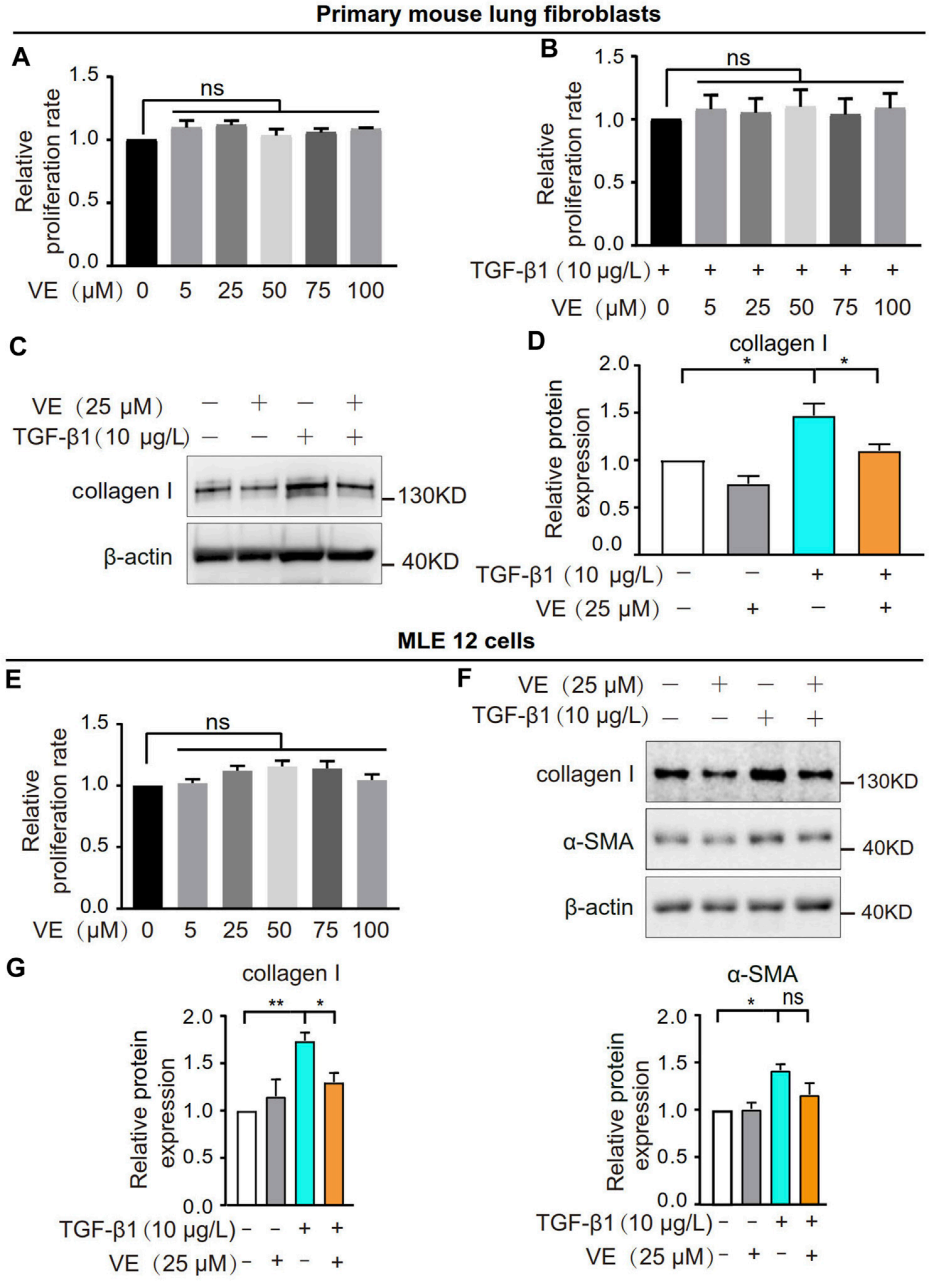
VE inhibited TGF-β1-induced fibrotic differentiation of fibroblasts and EMT of epithelial cells without influencing their proliferation *in vitro*. **(A,B)** Mouse primary lung fibroblasts were treated with VE **(A)** or cotreated with VE and TGF-β1 **(B)** as indicated, and the proliferation rate was examined by a thiazolyl blue tetrazolium bromide (MTT) kit. *N = 3*. **(C,D)** Expression level of collagen Ⅰ in mouse lung fibroblasts with the indicated treatments for 24 h was analyzed by immunoblot **(C)** and quantified by ImageJ **(D)**. *N = 4*. **(E)** Proliferation rate of mouse lung epithelial MLE 12 cells with the indicated treatments for 48 h was detected by MTT. *N = 3.* **(F,G)** MLE 12 cells were co-treated with TGF-β1 and VE for 24 h, and the expression levels of collagen Ⅰ and α-SMA were examined by immunoblot **(F)** and ImageJ was applied for quantitative analysis **(G)**. *N = 4*. ns, no significant differences. One-way ANOVA, *p < 0.05, **p < 0.01.

To explore whether VE promotes fibroblast activation and fibrotic differentiation *in vitro*, we treated primary mouse lung fibroblasts with VE and detected the proliferation rate and collagen I expression. VE did not alter the proliferation of fibroblasts or TGF-β1-induced myofibroblasts (Figures 3A, B) but decreased collagen I expression in myofibroblasts after TGF-β1 induction (Figures 3C, D). In addition, VE did not alter the proliferation rate but suppressed TGF-β1-induced expression of collagen I and α-SMA in MLE 12 cells (Figures 3E–G). Therefore, VE inhibits TGF-β1-induced fibrotic differentiation in fibroblasts and EMT in epithelial cells *in vitro*, whereas the suppression of fibroblast proliferation by VE *in vivo* may be associated with the proinflammatory microenvironment in BLM-PF lungs.

### 3.3 VE reduces the inflammatory response in bleomycin-induced fibrotic lungs and MLE 12 cells *in vitro*

**FIGURE 4:**
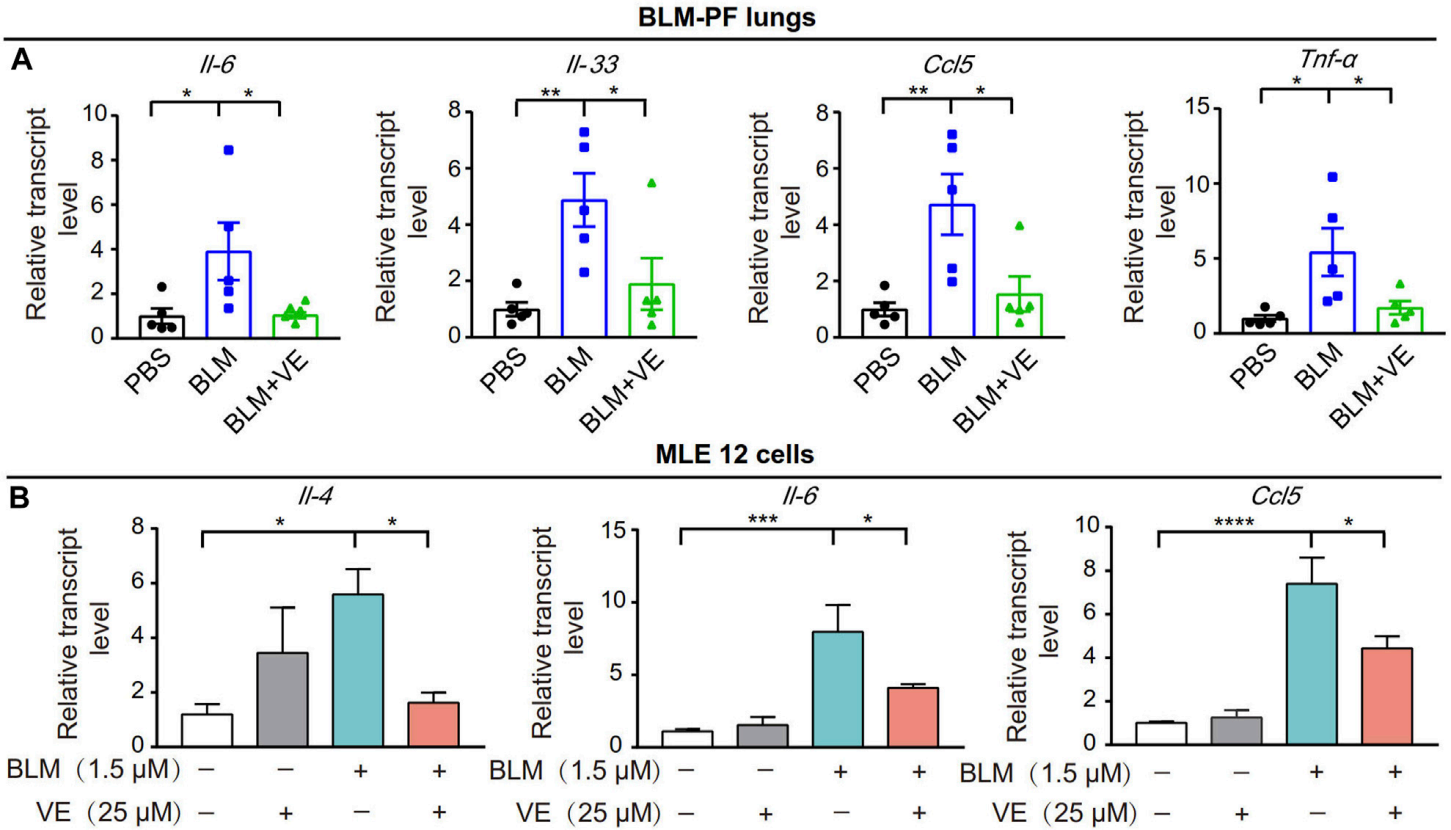
VE suppressed inflammation in BLM-PF mouse lungs and in cultured MLE 12 cells. **(A)** Transcription levels of *Il-6, Il-33, Ccl5* and *TNF-*α in mouse lungs on Day 14 post BLM injury were examined by real-time PCR. *n = 5/group*. **(B)** MLE 12 cells were co-treated with BLM and VE for 24 h and the transcription levels of *Il-*4, Il-*6* and *Ccl5* were detected by real-time PCR. *N = 4.* One-way ANOVA, *p < 0.05, **p < 0.01, ***p < 0.001, ****p < 0.0001.

Next, we tested whether VE affects the expression of proinflammatory cytokines in BLM-induced fibrotic lungs. We treated the corresponding animals with VE from Day 4 until Day 14 after BLM exposure. VE significantly decreased BLM-induced transcript levels of *Il-6*, *Il-33*, *Ccl5* and *Tnf-α* (Figure 4A). Moreover, VE downregulated the transcript levels of *Il-4, Il-6* and *Ccl5* in MLE 12 cells treated with BLM (Figure 4B). Thus, VE mitigates the proinflammatory microenvironment in BLM-PF lungs, which interacts with other biological processes in the lung and contributes to the attenuation of BLM-induced PF.

### 3.4 VE stabilizes iron homeostasis in BLM-induced fibrotic mouse lungs

**FIGURE 5:**
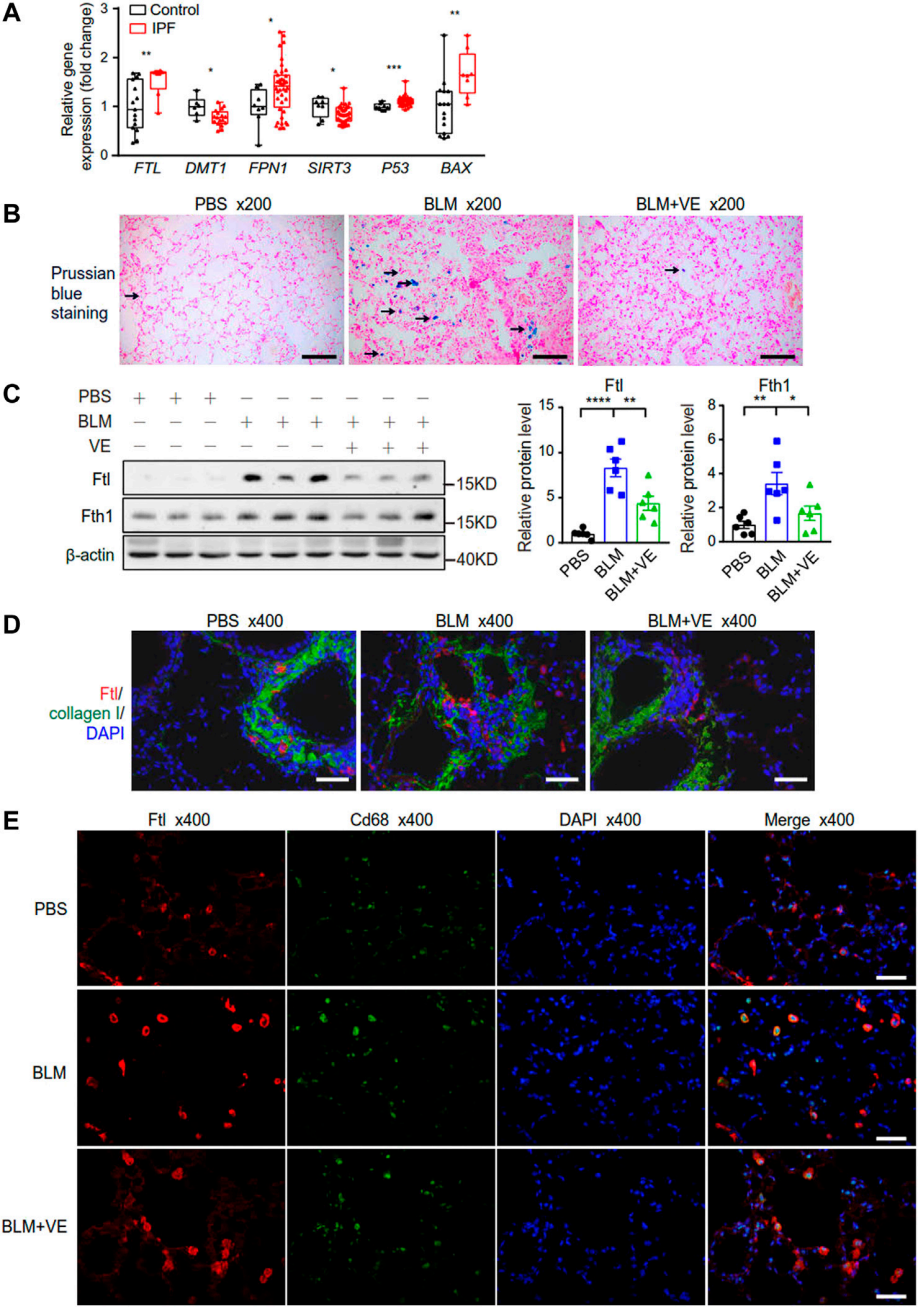
VE stabilized iron level and the expression of ferritin in BLM-induced mouse lungs. **(A)** Gene expression of the indicated genes in human lung tissues were downloaded from publicly accessible GEO database by array (FTL and BAX from the GSE10667 dataset with 15 normal lungs and 8 IPF lungs, DMT1 from the GSE24206 dataset with 6 normal lungs and 17 IPF lungs, FPN1, SIRT3, and P53 from the GSE53845 dataset with 8 normal lungs and 40 IPF lungs), and transformed to relative gene expression levels. Box and whiskers plots were drawn with bounds of the boxes and lines within the boxes representing the upper and lower quantiles and the median value. Mann-Whitney test. **(B)** Representative images of Pearl’s staining in mouse lung sections from the indicated groups. **(C)** Expression of Ftl and Fth1 (A, *n = 6/group*) in mouse lungs on Day 18 post BLM exposure was detected by immunoblot, and ImageJ was applied for quantitative analysis. One-way ANOVA. **(D,E)** Representative fluorescent immunostaining images of Collagen and Ftl **(D)**, and Cd68 and Ftl **(E)** on mouse lung sections from the indicated groups. *p < 0.05, **p < 0.01, ***p < 0.001, ****p < 0.0001.

Iron accumulation has been reported to promote PF (Ali et al., 2020; Zhu et al., 2021). First, we examined the transcript levels of some representative metabolic genes in the lung tissues of IPF patients through NCBI’s Gene Expression Omnibus database. For iron metabolism, expression of ferritin light chain (FTL) was increased in IPF patients compared with normal controls (Figure 5A), which was consistent with the reported iron accumulation in IPF lungs (Zhu et al., 2021). Reduced expression of divalent metal ion transporter (DMT1), which mediates iron release from phagosomes to the cytosol, and increased expression of ferroportin 1 (FPN1), which transport cellular iron out of the cell (Figure 5A), may be negative feedback due to increased iron levels. Mitochondrion-related protein sirtuin 3 (SIRT3), which is an NAD^+^-dependent protein deacetylase involved in the repair of mtDNA oxidative damage, was decreased in the IPF group (Figure 5A). Apoptosis marker proteins, tumor protein p53 (P53) and pro-apoptotic BCL2 associated X protein (BAX) exhibited increased expression in IPF lungs (Figure 5A). Therefore, the bioinformatics data demonstrated altered iron, mitochondrial metabolism, and increased apoptosis in the lungs of IPF patients. Next, to explore whether regulating iron homeostasis contributes to VE-mediated inhibition of PF, we performed Prussian blue staining and observed that VE decreased BLM-induced iron aggregation in BLM-PF mouse lungs on Day 18 post BLM instillation (Figure 5B). Further, VE treatment inhibited BLM-induced upregulation of Ftl and Fth1 (Figure 5C). Moreover, VE administration reduced the number of Ftl-positive cells around the fibrotic regions, and Ftl-positive macrophages in BLM-induced lung sections by immunostaining (Figures 5D, E).

**FIGURE 6:**
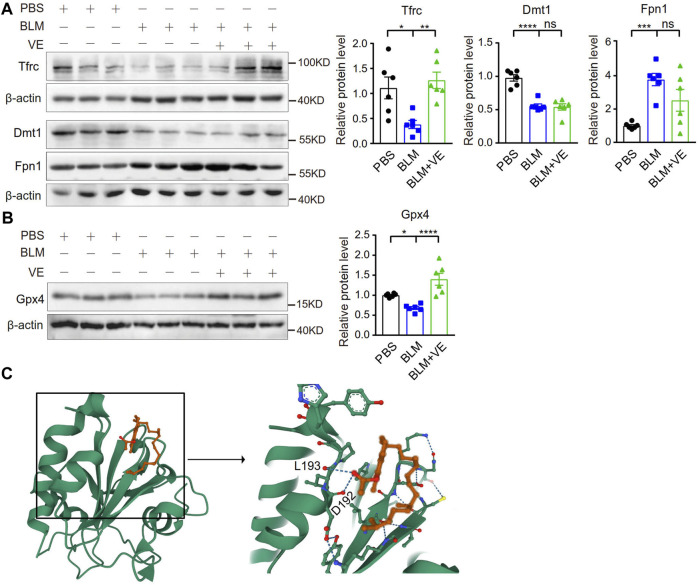
VE stabilized the expression of iron metabolism-related proteins and Gpx4 in BLM-induced mouse lungs. **(A,B)** Expression of the indicated iron-regulatory proteins (A, *n = 6/group*) and Gpx4 (B, *n = 6/group*) in mouse lungs on Day 18 post BLM exposure was detected by immunoblot, and ImageJ was applied for quantitative analysis. **(C)** Molecular docking study of Gpx4 and VE were performed by Autodock Vina 1.2.2 (http://autodock.scripps.edu/) and the three-dimensional structures of the binding pockets were shown. One-way ANOVA, *p < 0.05, ***p < 0.001, ****p < 0.0001. ns, no significant differences.

Importantly, VE significantly abrogated the BLM-induced reduction in the expression of Tfrc but did not alter the expression of Dmt1 and Fpn1 significantly (Figure 6A), indicating that VE partly normalized iron homeostasis. Iron generates ROS through the Fenton reaction. VE reversed BLM-induced suppression of Gpx4 (Figure 6B), an important antioxidant that prevents lipid peroxidation and regulates ferroptosis negatively. To uncover the mechanism how VE improve Gpx4 level, we analyzed the binding affinities and modes of interaction between GPX4 and VE by AutodockVina 1.2.2, a silico protein–ligand docking software. It showed that VE bound to GPX4 through visible hydrogen bonds with low binding energy of −6.1 kcal/mol, indicating stable binding (Figure 6C). These results showed that VE supplementation significantly stabilized iron-regulatory protein expression, and likely improved redox balance at least partly by regulation of Gpx4 in BLM-injured lungs.

### 3.5 VE does not chelate iron or alter cellular iron levels *in vitro*

**FIGURE 7:**
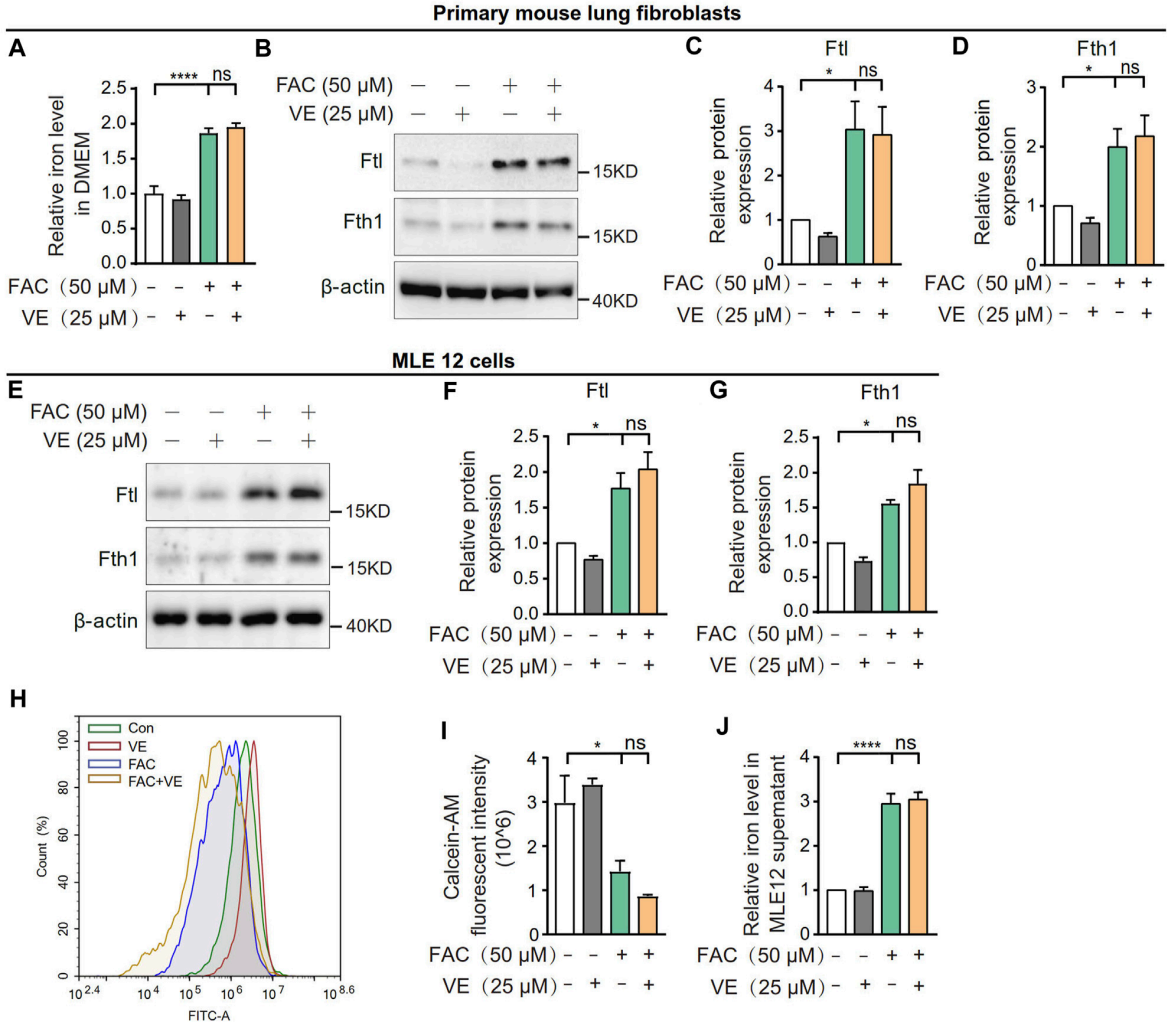
VE did not affect iron levels in cell culture medium or cellular iron. **(A)** Iron concentration in DMEM medium supplemented with FAC and VE for 24 h was detected. *N = 11*. **(B–D)** Mouse pulmonary fibroblasts were co-treated with FAC and VE for 24 h. The expression of Ftl and Fth1 was detected by immunoblot **(B)** and ImageJ was applied for quantitative analysis **(C,D)**. *N = 7*. **(E–I)** MLE 12 cells were co-treated with FAC and VE for 24 h. The expression of Ftl and Fth1 was examined by immunoblot **(E)** and quantified by ImageJ **(F,G)**. *N = 3*. MLE 12 cells with the indicated treatments were stained with calcein-AM, a fluorescent reagent that binds to ferrous and ferric iron, and the fluorescent intensity was measured by flow cytometry **(H,I)**. *N = 3*. The iron concentration in MLE 12 cell supernatant was detected by a tissue iron assay kit (A039-2-1; Nanjing Jiancheng Bioengineering Institute, Nanjing, China) **(J)**. *N = 6*. ns, no significant differences. One-way ANOVA statistical analysis, *p < 0.05, ****p < 0.0001.

To explore whether VE chelates iron directly, we first measured the effect of VE on the iron level in DMEM supplemented with ammonium ferric citrate (FAC). VE did not decrease the iron level in the medium in the presence of FAC (Figure 7A). Furthermore, VE did not decrease the FAC-induced increase in the expression of Ftl and Fth1 in mouse primary lung fibroblasts (Figures 7B–D) or MLE 12 cells (Figures 7E–G), nor did VE alter FAC-induced iron levels which is correlated inversely to the fluorescence intensity of calcein acetoxymethyl ester (calcein-AM) in MLE 12 cells (Figures 7H, I) or iron levels in the cell supernatant (Figure 7J). Similarly, the water-soluble VE analog Trolox did not significantly affect the expression of Ftl and Fth1 in FAC-treated MLE 12 cells (Supplementary Figure S1A–C). Thus, VE does not chelate iron directly or regulate cellular iron levels *in vitro*, suggesting that the stabilization of iron metabolism by VE in BLM-induced fibrotic lungs is associated with the effects of VE on the pulmonary microenvironment.

### 3.6 VE normalizes mitochondrial protein expression and cell apoptosis in BLM-induced fibrotic lungs

**FIGURE 8:**
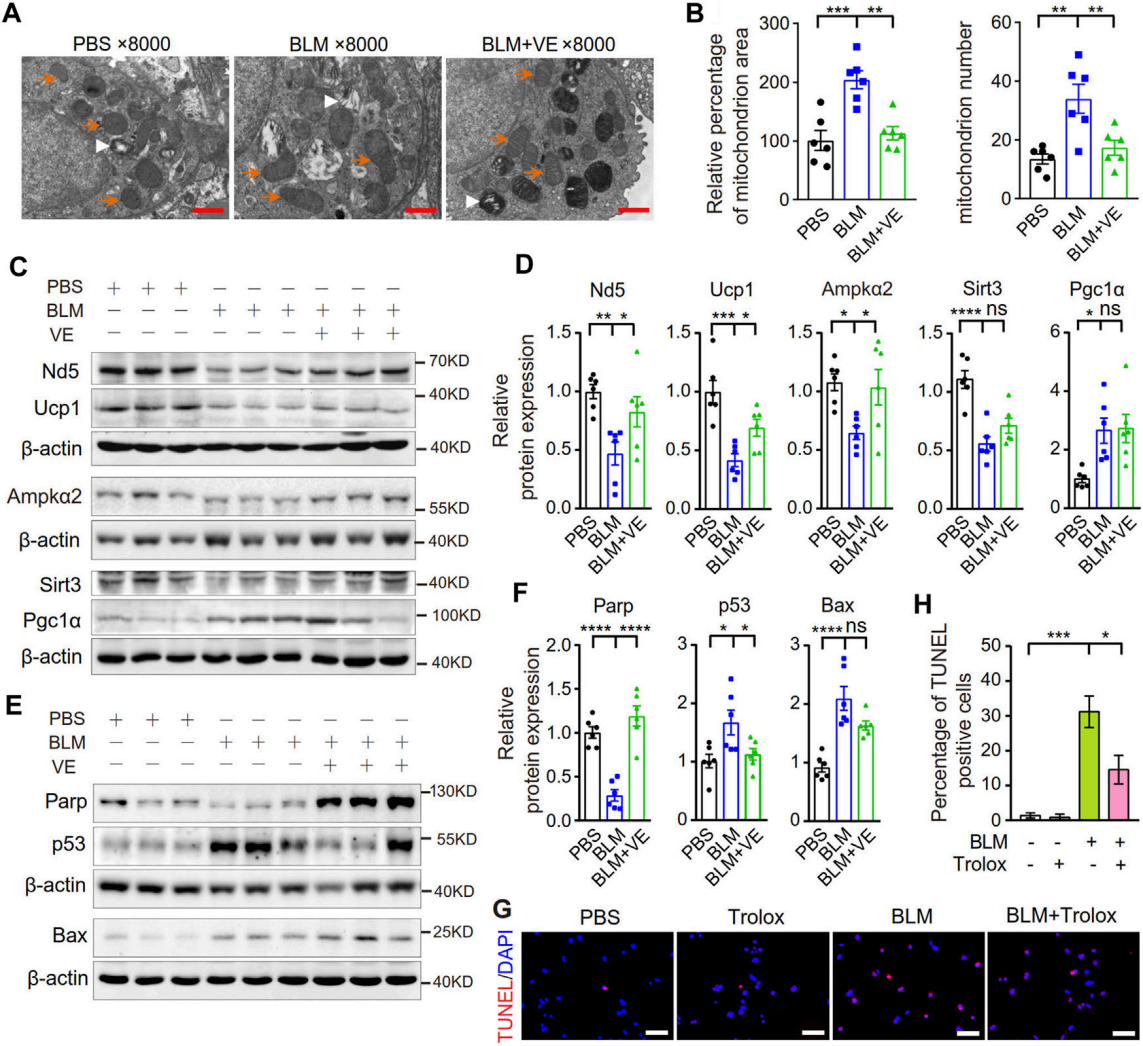
VE improved mitochondrial morphology and stabilized the expression of mitochondria-related proteins and apoptosis-related proteins. **(A)** Representative images of mitochondria in AECIIs from mouse lungs on Day 18 post BLM injury under transmission electron microscope were shown. Orange arrows indicate some of the mitochondria, and white arrowheads indicate some of the lamellar bodies. Scale bars: 1 μm. **(B)** Mitochondrial area was analyzed by ImageJ and mitochondrial number was counted in AECIIs (*n = 6/group*). **(C–F)** Expression levels of mitochondrial-related proteins (C and D, *n = 6/group*) and apoptosis-related proteins in mouse lungs on Day 18 post BLM instillation were examined by immunoblot (E and F, *n = 6/group*) and quantified by ImageJ. **(G,H)** MLE 12 cells were cotreated with BLM (25 μM) and Trolox (200 μM) for 24 h, stained by a TMR (red) TUNEL cell apoptosis detection kit (G1502, Servicebio) (G, Scale bars: 100 μm) and the percentage of TUNEL positive cells among all the cells in the microscopic images were calculated **(H)**. *N = 3*. ns, no significant differences. *p < 0.05, **p < 0.01, ***p < 0.0001, and ****p < 0.0001 by One-way ANOVA.

Excess ROS cause mitochondrial structural damage and dysfunction (Syed et al., 2021). It has been reported that the mitochondrial structure in alveolar epithelial type II cells (AECIIs) of BLM-induced fibrotic lungs or in cultured lung epithelial cells with BLM addition was damaged (Yu et al., 2018; Shaghaghi et al., 2021). To detect whether VE improves mitochondrial structural injury caused by BLM, we observed the mitochondrial ultrastructure and examined the expression of mitochondrion-related proteins in BLM-PF lungs treated with VE. Compared with PBS control lungs, mitochondria in BLM-induced lungs were swollen and accumulated. VE treatment significantly reduced BLM-induced mitochondrial area and number in the AECIIs of fibrotic lungs (Figures 8A, B). Importantly, as shown in Figures 8C, D, some proteins related to mitochondrial structure and function, including Nd5, Ucp1, Ampkα2, and Sirt3, in BLM-instilled lungs were decreased. These changes were significantly normalized by VE treatment, except for Sirt3, which was slightly altered. The mitochondrial gene Nd5 encodes a complex I subunit, and Ucp1 causes electron transport and ATP uncoupling in mitochondrial oxidative respiration. AMP-activated protein kinase (Ampk) is a key molecule that regulates bioenergy metabolism, and its activity is lower in fibrotic regions associated with metabolically active and apoptosis-resistant myofibroblasts in the BLM-PF mouse model (Rangarajan et al., 2018). Sirt3 is an NAD^+^-dependent protein deacetylase that plays a role in the repair of mtDNA oxidative damage. Alveolar epithelial cells of IPF patients exhibited Sirt3 deficiency, and the overexpression of Sirt3 ameliorated asbestos-induced PF (Cheresh et al., 2021). However, Pgc-1α expression was significantly increased in BLM-PF lungs, which coincides with the accumulation of mitochondria. VE reduced mitochondrial numbers in BLM-PF lungs but did not significantly alter Pgc-1α expression, indicating the upregulation of Pgc-1α signaling by VE.

Mitochondrial structural damage and dysfunction are prone to cause mitochondrial death, which is an important endogenous pathway leading to cell apoptosis. BLM-injured lungs showed lower Parp1 levels, indicating more cleavage of Parp1; and higher levels of the apoptosis-related proteins p53 and Bax, and VE suppressed these alterations significantly or in a trend (Figures 8E, F). Consistently, Trolox inhibited the BLM-induced increase in the percentage of TUNEL-positive cells by 56% (Figures 8G, H), also alleviated BLM-reduced proliferation rate of lung fibroblasts (Supplementary Figure S2) Collectively, these results demonstrated that VE improved mitochondrial structural damage and dysfunction and inhibited apoptosis in fibrotic mouse lungs, which contributed to the attenuation of PF.

Importantly, we evaluated the effects of VE on mice by administration of VE alone to the mouse instilled with PBS. It showed that VE alone does not affect the body weight (Supplementary Figure S3A), parameters in fibrosis (Supplementary Figures S3B-E), the levels of proteins included in iron metabolism (Supplementary Figure S4A), Gpx4 and mitochondrial metabolism (Supplementary Figure S4B) and apoptosis (Supplementary Figure S4C) in comparison to that of the control mice. These results confirmed that VE has few side effects on normal mice.

## 4 Discussion

VE possesses anti-inflammatory, anticancer and antimutagenic properties (Jin et al., 2014; Tang et al., 2016). There has been only a few reports about the attenuation of experimental PF caused by different inducers besides BLM, such as amiodarone, radiation, melphalan, and hexavalent chromium, however, all of the identified molecular mechanisms are limited to antioxidation and the inhibition of inflammation (Card et al., 2003; Bese et al., 2007; Hemmati et al., 2008; Wigenstam et al., 2009). This study observed the improvement in BLM-induced lung pathology and survival decrease of experimental animals by VE, confirming a potent mitigation effect of VE in severe PF. For the first time, we uncovered and evidenced the novel mechanisms of VE against PF include suppressing the pathological activation and fibrotic differentiation of lung fibroblasts and epithelial-mesenchymal transition, and importantly, the improvement of metabolisms of iron and mitochondria, and apoptosis in BLM-induced fibrotic lungs. We also investigated the molecular functional mechanisms of VE *in vitro*. These results deepen our understanding of the protective effects of VE against PF, which may be underestimated before.

Oxidative stress and inflammation play important roles in IPF and experimental PF models (Bringardner et al., 2008). VE concentrations in the hamster lungs increase significantly after intratracheal BLM administration, and diet-deficiency of VE increases levels of lipid peroxide in the hamster lungs at a very early stage post BLM treatment (Kato et al., 1990) Administration of α-tocopherol increases glutathione level and catalase activity, and decreased malondialdehyde level in bleomycin-induced fibrotic lungs of rats (Deger et al., 2007). It has been reported that VE reduces the release of proinflammatory cytokines by limiting the production of ROS and ROS-mediated signal transduction, preventing chronic inflammation, such as liver fibrosis (Singh et al., 2005; Zingg, 2007; Calder et al., 2009). Consistently, VE deficiency enhances pulmonary inflammatory response and oxidative stress induced by single-walled carbon nanotubes in mice (Shvedova et al., 2007). Tocopherol diet normalizes belomycin-induced increase in reactive oxygen and nitrogen species, and mitigated inflammation progression, demonstrated by reduced macrophage activation and function, and limited enzymes and cytokines involved in pro-inflammation in mouse lungs (Shi et al., 2013). In a clinical trial, the supplementation with a combination of vitamins D, C and E remarkably reduced the serum levels of protein carbonyl, high-sensitivity C-Reactive Protein and TGF-β in IPF patients (Yavari et al., 2022). Consistently, this study confirmed that VE significantly upregulated Gpx4 expression and suppressed the transcript levels of inflammatory cytokines in BLM-induced fibrotic lungs and MLE 12 cells *in vitro*. Thus, inhibition of the oxidative stress and inflammatory response contribute to the protective effects of VE against PF.

Iron is an essential trace element for multicellular organisms and most microorganisms. It is a catalytic component of some enzymes that mediate many redox reactions that are critical for energy production and intermediate metabolism (Ganz and Nemeth, 2015). Iron also plays an important role in the regulation of the cell cycle, cell division and cell metabolism (Heath et al., 2013). Therefore, the maintenance of iron homeostasis in organs is crucial for their normal function in the organism. Iron catalyzes electron exchange and disturbs iron homeostasis through oxidative stress in the lung, resulting in lung tissue damage. BLM-induced PF and decreased lung function in experimental mice are related to pulmonary iron accumulation (Ali et al., 2020). Alpha-tocopherol decreased BLM-triggered pulmonary iron concentrations in rats (Ertekin et al., 2007). In this study, VE treatment significantly reduced BLM-induced iron aggregation and normalized the expression of ferritin and iron-regulatory proteins, indicating that VE improves pulmonary iron level as well as metabolism. It has been reported that iron promotes the proliferation of pulmonary fibroblasts in BLM-induced fibrotic lungs and *in vitro* (Zhu et al., 2021). Thus, VE reduces the total number of fibroblasts and the number of proliferating fibroblasts in BLM-induced lungs, which may be mediated by reducing pulmonary iron levels. However, *in vitro*, VE had no significant effect on FAC-induced expression of ferritin, cellular iron, or cell supernatant iron levels or on the proliferation level in TGF-β1-induced myofibroblasts. These results indicate that VE does not directly chelate iron or directly affect fibroblast proliferation under physiological conditions, but VE reduces the pathological increase in pulmonary iron levels *in vivo*, which is likely dependent on less inflammatory microenvironment in the fibrotic lung, thereby reducing the proliferation of pulmonary fibroblasts *in vivo*. In addition, VE decreased the number of myofibroblasts and the expression of the main extracellular matrix component collagen I in BLM-PF lungs, and it also reduced TGF-β1-induced fibrotic differentiation in lung fibroblasts and EMT in epithelial cells *in vitro*. These effects do not seem to be dependent on iron levels, and they may be related to VE-reduced cellular ROS production or secretion of proinflammatory factors.

Mitochondrial dysfunction has been found in pulmonary AECIIs, fibroblasts, and macrophages in IPF patients, which increases sensitivity to fibrosis (Bueno et al., 2020). Mitochondrial dysfunction results from mitochondrial damage, which is caused by adverse environmental conditions, including excessive mitochondrial reactive oxygen species (mtROS), changes in mitochondrial morphology, disturbed oxidative phosphorylation, impaired energy production, alterations in metabolic pathways (i.e., fatty acid β-oxidation) (Nunnari and Suomalainen, 2012; Cloonan and Choi, 2016; Bueno et al., 2020). Alpha-tocopherol is crucial for mitochondrial integrity, as profound deficiency of α-tocopherol causes mitochondrial degeneration and leads to necrosis of skeletal muscle in rats (Van Vleet et al., 1968). VE has been reported to play an important role in reducing mitochondrial damage and mitochondrial oxidative dysfunction mediated by clearing ROS (Napolitano et al., 2019), or prevent the reduction in the electron transfer rate in aged mice (Navarro et al., 2005; Polyak et al., 2018). For instance, VE restored the activity and expression of the third subunit of cytochrome-c oxidase in lung mitochondria and bronchial epithelia, and improved mitochondrial ultrastructure of bronchial epithelia in an ovalbumin-challenged allergic asthma murine model (Mabalirajan et al., 2009). VE fully prevented the brain death of zebrafish in the rotenone-induced mitochondrial complex I disease model (Polyak et al., 2018). However, VE did not prevent mitochondrial dysfunction in amiodarone-induced pulmonary fibrosis in hamster (Card et al., 2003). The potential reasons for the divergent conclusions include different action mechanisms of the drugs, outcome measures, species, and dietary conditions. In this study, AECIIs in BLM-induced lungs showed mitochondrial swelling, which was inhibited by VE. Importantly, the total mitochondrial numbers in AECIIs were increased in BLM-induced lungs, and VE treatment significantly reduced mitochondrial numbers, suggesting that VE suppressed the accumulation of mitochondria with impaired structure and function. Notably, we found that the expression of mitochondrial structure- and function-related proteins (Ampkα2, Ucp1, Nd5, and Sirt3) was downregulated by BLM exposure, and this change was restored by VE treatment significantly or in a trend, strongly suggesting that VE improves the structure and function of BLM-damaged mitochondria.

Mitochondrial damage and dysfunction can lead to cell senescence and even cell death. BLM-induced fibrotic mouse lungs exhibited higher levels of apoptosis than the controls (Sul et al., 2022) and ferroptosis of epithelial cells was included (Pei et al., 2022). In this study, altered expression levels of the apoptosis-related proteins Parp1, p53, and Bax in BLM-PF lungs indicated increased cell apoptosis, which was restored by VE significantly or in a trend. Consistently, Trolox mitigated BLM-induced apoptosis in pulmonary epithelial cells and inhibits fibroblast death from BLM injury *in vitro*. As VE compensates for Gpx4 loss by protecting hepatocytes against deleterious lipid peroxidation (Carlson et al., 2016), the normalization of BLM-reduced Gpx4 levels by VE is suggested to contribute to the inhibition of pulmonary cell death in our study. Similarly, VE protects against cigarette smoke extract-induced cell apoptosis in a dose- and time-dependent manner in mouse embryonic cells (Chen et al., 2011). The inhibitory effect of VE on apoptosis in fibrotic lungs and pulmonary epithelial cells may involve improvements in mitochondrial structure damage and dysfunction.

In conclusion, in addition to confirming the strong inhibitory effect of VE on PF, this study demonstrated novel mechanisms by which VE protects against PF, including stabilizing iron metabolism in an experimental mouse model, improving mitochondrial structure, and suppressing the activation and fibrotic differentiation of fibroblasts and EMT, besides the known weakening of inflammatory response and strong antioxidant ability. Considering the safety and efficacy of VE, it is a potential therapeutic for treating PF and is worth further clinical trials to determine its clinical application. The regulation in metabolisms of iron and mitochondria by VE that were delineated in this work also sheds light on the therapeutics of other diseases with similar metabolic disorders.

## Data Availability

The original contributions presented in the study are included in the article/Supplementary Material, further inquiries can be directed to the corresponding authors.
