# Supplementary material for: Vitamin E stabilizes iron and mitochondrial metabolism in pulmonary fibrosis
Source: Front Pharmacol. 2023 Dec 6;14:1240829. doi: 10.3389/fphar.2023.1240829 (PMC10731373; doi:10.3389/fphar.2023.1240829)
Supplement: Supplementary file 1 [file DataSheet1.pdf]

Online Data Supplement

Manuscript title: Vitamin E Stabilizes Iron and Mitochondrial Metabolism in Pulmonary Fibrosis

Authors: Jing Chang, Jiahui Wang, Beibei Luo, Weihao Li, Ziyue Xiong, Chaoqi Du, Xue Wang, Yuejiao Wang, Jingya Tian, Shuxin Li, Yue Fang, Longjie Li, Jing Dong, Ke Tan, Yumei Fan, Pengxiu Cao

Primary antibodies applied in immunoblot analysis

The following primary antibodies were used: anti-collagen I (1:500, GB01022-1, Servicebio, Wuhan, China), anti-smooth muscle actin α 2 (α-SMA, 1:1000, ARG66381, Arigo, Hsinchu, China), anti-ferritin light chain (Ftl, 1:1000, ab69090, Abcam, Cambridge, USA), anti-ferritin heavy polypeptide 1 (Fth1, 1:1000, ab65080, Abcam), anti-Tfrc (1:1000, K004269P, Solarbio), anti-Dmt1 (1:1000, K000336P, Solarbio), anti-Fpn1 (1:500, A14884, ABclonal, Wuhan, China), anti-glutathione peroxidase 4 (Gpx4, 1:1000, ab125066, Abcam), anti-AMP-activated protein kinase alpha 2 catalytic subunit (Ampkα2, 1:1000, ab97275, Abcam), anti-peroxisome proliferator activated receptor gamma coactivator 1 alpha (Pgc-1α, 1:1000, AF5395, Affinity Biosciences, Liyang, China), anti-mitochondrial uncoupling protein 1 (Ucp1, 1:500, DF7720, Affinity Biosciences), anti-NADH dehydrogenase subunit 5 (Nd5, 1:500, K006368P, Solarbio), anti-sirtuin 3 (Sirt3, 1:500, K005158P, Solarbio), anti-p53 (1:1000, ab90363, Abcam), anti-BCL2-associated X protein (Bax, 1:500, K1593P, Solarbio), anti-poly (ADP-ribose) polymerase family member 1 (Parp1, 1:1000, AF5264, Beyotime, Shanghai, China), and anti-β-actin (1:1000, GB12001, Servicebio).

Primer sequences for quantitative real-time PCR

The following gene primer pairs were applied: *collagen type I alpha 1* (*Col1a1*) forward, CTTCACCTACAGCACCCTTG, *Col1a1* reverse, CCAATGTCTAGTCCGAATTCCT; *Il-6* forward, TGATTGTATGAACAACGATGATGC, *Il-6* reverse, GGTACTCCAGAAGACCAGAGGAAA; *Il-33* forward, CTTTCCTTTTCTCTGCCTTGAGTC, *Il-33* reverse, GAAATGGACCCTCTCTAAAGCAAA; *Ccl5* forward, GCTGCTTTGCCTACCTCTCC, *Ccl5* reverse, TCGAGTGACAAACACGACTGC; *Tnf-α* forward, GACGTGGAACTGGCAGAAGAG, *Tnf-α* reverse, TTGGTGGTTTGTGAGTGTGAG; *Il-4* forward, TCAACCCCCAGCTAGTTGTC, *Il-4* reverse, TGTGACCTCGTTCAAAATGC; and *β-actin* forward, GGCTGTATTCCCCTCCATCG, *β-actin* reverse, CCAGTTGGTAACAATGCCATGT.

Supplemental Figure 1

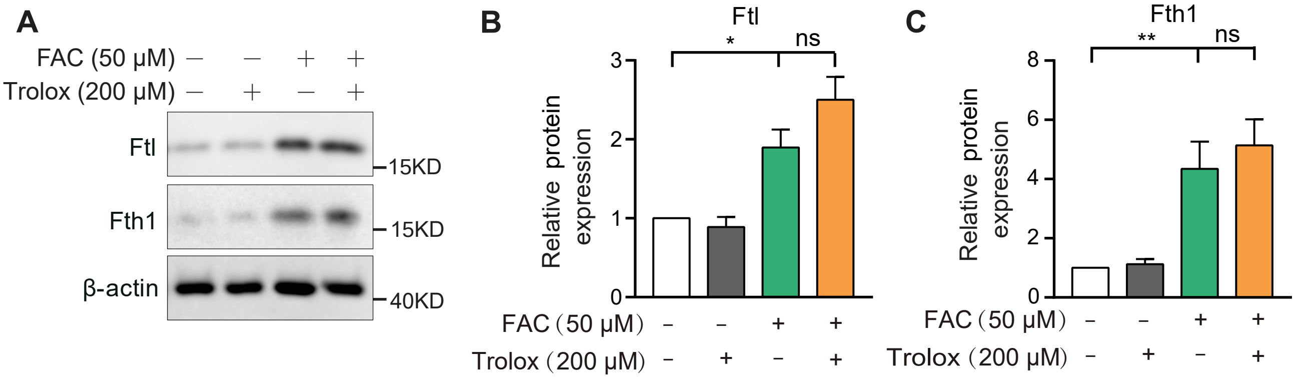

Supplemental Figure 1. Induced-expression of Ftl and Fth1 by FAC in MLE 12 was not affected significantly by Trolox. (**A**-**C**) MLE 12 cells were co-treated with FAC and Trolox for 24 h and the expression of Ftl and Fth1 was detected by immunoblot (**A**) and quantified by ImageJ (**B** and **C**). *N* = *6*. ns, no significant difference. * $p < 0.05$, ** $p < 0.01$ by One-way ANOVA.

Supplemental Figure 2

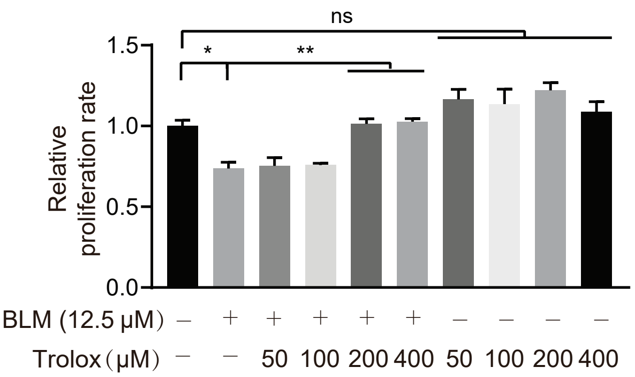

Supplemental Figure 2. Trolox inhibited BLM-induced cell death in lung fibroblasts in vitro. Mouse primary lung fibroblasts were cotreated with BLM and Trolox as indicated, and the proliferation rate was examined by a thiazolyl blue tetrazolium bromide (MTT) kit. *N = 3*. One-way ANOVA, * P<0.05, ** P<0.01, ns, no significant differences.

Supplemental Figure 3

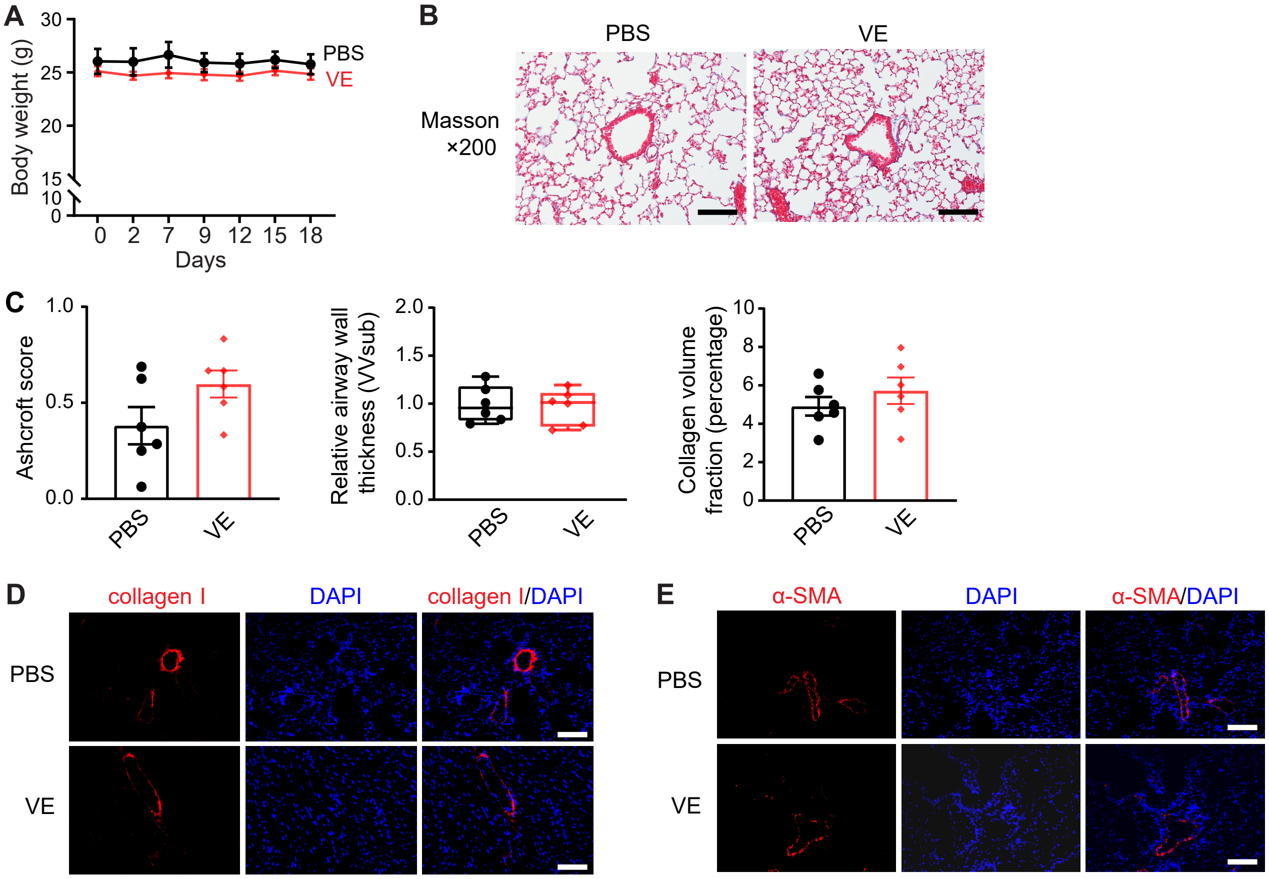

Supplemental Figure 3. VE alone did not affect fibrosis-related features in mouse lungs significantly. VE treatment started from Day 7 till harvest on Day 18 post PBS instillation. (**A**) Changes in the body weight during the time-course post PBS instillation. (**B**) Representative images of Masson-stained lung sections as indicated. Scale bars, 100 μm. (**C**) Ashcroft score, relative airway wall thickness, and collagen volume fraction were analyzed based on Masson-stained images. (**D** and **E**) Representative immunofluorescent staining images of collagen I (**D**) and α-SMA (**E**) in mouse lung sections. Scale bars, 100 μm. *n = 6/group*. Two-way ANOVA (**A**), and *t*-test (**C**) were conducted and no significant differences were observed.

Supplemental Figure 4

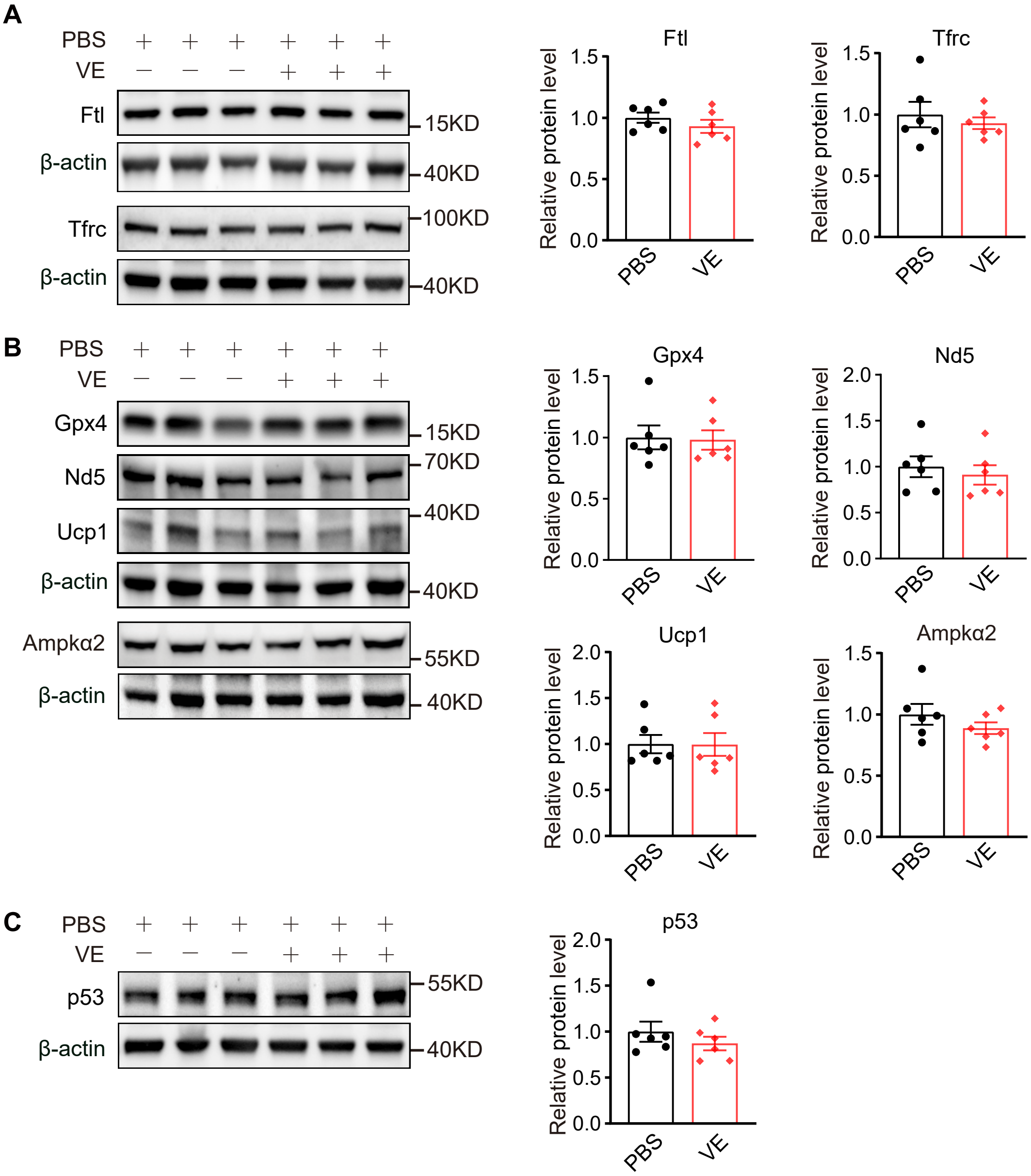

Supplemental Figure 4. VE alone did not affect expression levels of representative proteins in metabolisms of iron and mitochondria, and apoptosis in mouse lungs significantly. (**A-C**) Expression levels of representative proteins in iron metabolism (**A**), Gpx4 and mitochondrial metabolism (**B**), and apoptosis (**C**) in mouse lungs on Day 18 post PBS instillation were examined by immunoblot, and ImageJ was utilized for quantitative analysis. *n = 6/group*. *T*-test was conducted and no significant differences were observed.
